# Non-Specific Effects of Bacillus Calmette-Guérin: A Systematic Review and Meta-Analysis of Randomized Controlled Trials

**DOI:** 10.3390/vaccines11010121

**Published:** 2023-01-04

**Authors:** Gerhard Trunk, Maša Davidović, Julia Bohlius

**Affiliations:** 1 Independent Researcher, 3007 Bern, Switzerland; 2 Department of Epidemiology and Public Health, Swiss Tropical and Public Health Institute, 4123 Allschwil, Switzerland; 3 University of Basel, 4001 Basel, Switzerland; 4 Graduate School of Health Sciences, University of Bern, 3012 Bern, Switzerland; 5 Department for Education and Training, Swiss Tropical and Public Health Institute, 4123 Allschwil, Switzerland; 6 Institute of Social and Preventive Medicine, University of Bern, 3012 Bern, Switzerland

**Keywords:** BCG, vaccine, non-specific effects, trained immunity, respiratory infection, COVID-19, pandemic preparedness

## Abstract

Background: Vaccines induce antigen-specific immunity, which provides long-lived protection from the target pathogen. Trials from areas with high incidence rates for infectious diseases indicated that the tuberculosis vaccine Bacillus Calmette-Guérin (BCG) induces in addition non-specific immunity against various pathogens and thereby reduces overall mortality more than would have been expected by just protecting from tuberculosis. Although recent trials produced conflicting results, it was suggested that BCG might protect from non-tuberculosis respiratory infections and could be used to bridge the time until a specific vaccine against novel respiratory diseases like COVID-19 is available. Methods: We performed a systematic search for randomized controlled trials (RCTs) published between 2011 and December 9^th^, 2022, providing evidence about non-specific effects after BCG vaccination, assessed their potential for bias, and meta-analyzed relevant clinical outcomes. We excluded RCTs investigating vaccination with an additional vaccine unless outcomes from a follow-up period before the second vaccination were reported. Results: Our search identified 16 RCTs including 34,197 participants. Vaccination with BCG caused an estimated 44% decrease in risk for respiratory infections (hazard ratio (HR) 0.56, 95% confidence interval (CI) 0.39–0.82) with substantial heterogeneity between trials (I^2^ = 77%). There was evidence for a protective effect on all-cause mortality of 21% if follow-up was restricted to one year (HR 0.79, 95% CI 0.64–0.99). We did not find evidence for an effect when we considered longer follow-up (HR 0.88, 95% CI 0.75–1.03). Infection-related mortality after BCG vaccination was reduced by 33% (HR 0.67; 95% CI 0.46–0.99), mortality for sepsis by 38% (HR 0.62, 95% CI 0.41–0.93). There was no evidence for a protective effect of BCG vaccination on infections of any origin (HR 0.84, 95% CI 0.71–1.00), COVID-19 (HR 0.88, 95% CI 0.68–1.14), sepsis (HR 0.78, 95% CI 0.55–1.10) or hospitalization (HR 1.01, 95% CI 0.91–1.11). Conclusions: According to these results, depending on the setting, vaccination with BCG provides time-limited partial protection against non-tuberculosis respiratory infections and may reduce mortality. These findings underline BCG’s potential (1) in pandemic preparedness against novel pathogens especially in developing countries with established BCG vaccination programs but limited access to specific vaccines; (2) in reducing microbial infections, antimicrobial prescriptions and thus the development of antimicrobial resistance. There is a need for additional RCTs to clarify the circumstances under which BCG’s non-specific protective effects are mediated.

## 1. Introduction

Bacillus Calmette-Guérin (BCG) is an attenuated bacterial vaccine developed against the respiratory infectious disease tuberculosis. According to the current World Health Organization (WHO) policy, it is mainly used in tuberculosis-endemic countries to protect children against the disease [1,2]. Besides its disease-specific protective effects, several studies indicate that BCG might also protect against non-tuberculosis pathogens [3]. Observational studies and trials conducted in newborns in developing countries reported significant reductions in mortality after BCG vaccination, which cannot be solely explained by protection from tuberculosis [4,5,6]. However, some trials mainly from high-income countries could not confirm these results [7,8]. The mechanisms behind these non-specific effects (NSE) of BCG are incompletely understood but likely involve multifactorial effects on the innate and adaptive immune response. One described mechanism is trained immunity: a state of innate immune memory that can be induced by vaccination, leading to epigenetic and metabolic reprogramming of innate immune cells such as monocytes and natural killer cells [9,10,11]. Moreover, emergency granulopoiesis and heterologous T cell reactivity have been described [12,13]. 

The sudden occurrence of “Coronavirus disease 2019” (COVID-19), a severe and potentially lethal respiratory disease caused by the novel coronavirus SARS-CoV-2, and its spread as a global pandemic triggered intensive scientific efforts to develop a SARS-CoV-2 specific vaccine, which was perceived as the most effective way out of the pandemic. To bridge the time until a SARS-CoV-2 specific vaccine was available, it had been suggested to evaluate BCG’s potential in providing non-specific protection, in particular in people with a high risk for COVID-19 [14]. More than 40 randomized controlled trials (RCTs) with approximately 75,000 participants worldwide were initiated [15]. However, the fast development of several COVID-19-specific vaccines and the launch of national COVID-19 vaccination programs severely hampered the recruitment of COVID-19 naïve and non-vaccinated participants for BCG-COVID-19 RCTs [16]. Consequently, these trials are way behind schedule and it is questionable whether the effect of a BCG vaccination alone on COVID-19 can be answered by them. 

Although COVID-19-specific vaccines are available by now, there is a need for an assessment of BCG’s NSE and its potential as a bridge vaccination due to (1) its wide availability, especially in developing countries which often suffer from timely access to specific vaccines and medical supplies; (2) its potential to activate the immune system against various pathogens, like emerging vaccine resistant SARS-CoV-2 immune escape mutants or novel pathogens in future pandemics. Moreover, BCG-mediated trained immunity may reduce microbial infections in general, thereby lessening drug prescriptions and the development of antimicrobial resistance; it may also reduce the risk for all-cause mortality.

We aimed to evaluate the effects of BCG vaccination on the risk for non-tuberculosis respiratory infections, COVID-19, non-tuberculosis infections of any origin, sepsis, mortality, and hospitalizations. For this purpose, we systematically identified RCTs published between 2011 and 9 December 2022, which compared the effects of BCG vaccination against no vaccination, assessed their potential for bias, and quantified the estimated effects by meta-analysis.

## 2. Materials and Methods

We performed a systematic review and meta-analysis of RCTs reporting NSE of vaccination with BCG. The study protocol is registered in PROSPERO (CRD 42021255017).

### 2.1. Data Sources and Search Strategy

A systematic literature search was conducted in four electronic databases (Medline via Pubmed and Ovid, Embase, Cochrane CENTRAL, Living Evidence on COVID-19) and three electronic clinical trial registers (Cochrane COVID-19 Study Register, clinical-trials.gov, and WHO International Clinical Trials Registry Platform) from 1 January 2011 to 9 December 2022 (Table S1). In addition, we screened the reference lists of included literature and relevant reviews for additional references. Literature published in English was considered. Duplicate records were removed with Deduklick [17]. The search strategy is described in detail in Table S2.

### 2.2. Study Selection and Eligibility Criteria

We included studies that met the following criteria: (1) RCTs; (2) conducted among children and/or adults; (3) comparing BCG vaccination with placebo/no vaccination; (4) reporting effects of BCG vaccination on non-tuberculosis related respiratory infections, non-tuberculosis related infections of any origin, non-tuberculosis related sepsis, non-tuberculosis related mortality (due to all cause, infectious diseases, respiratory infections, sepsis); non-tuberculosis related hospitalizations (due to all cause, infectious diseases, respiratory infections, sepsis). Trials conducted in participants with interfering comorbidities, e.g., patients with bladder cancer for which BCG is used as a therapeutic option, and trials investigating combinations of interventions were excluded (Table S3). If trial participants received additional vaccines during the follow-up of the study, we included only the period before the second vaccination. We excluded trials in which the timing of a different second vaccination during the follow-up period was unclear. Two reviewers (GT, MD) independently screened titles and/or abstracts of studies retrieved to identify studies that potentially met the inclusion criteria. Full texts of potentially eligible studies were independently assessed for eligibility by two review authors (GT, MD). In case of disagreement between reviewers over the eligibility of particular studies, a third reviewer (JB) was consulted. We used the Rayyan web application for screening [18]. 

### 2.3. Data Extraction

Study characteristics and outcome data were recorded by one reviewer (GT) using a standardized data collection form. Another reviewer checked the data (MD). If methods or study design were described in several publications, all publications were used to inform data extraction. If publications with additional analyses for a given trial were available, the publication providing most information was considered.

### 2.4. Risk of Bias Assessment

Two reviewers (GT, MD) evaluated independently the risk of bias for each included study using the Cochrane Collaboration’s tool for assessing the risk of bias [19]. Any disagreements over the risk of bias in particular studies were resolved by consultation of a third reviewer (JB). Studies were evaluated based on the following criteria: random sequence generation, allocation concealment, blinding of participants/researchers, blinding of outcome assessment, incomplete outcome data, selective reporting, and other bias due to problems not covered by the previous criteria [20]. 

### 2.5. Data Analysis

We assessed the following outcomes: non-tuberculosis respiratory infections; COVID-19; infections of any origin; sepsis; mortality due to all causes, infectious diseases, sepsis; and hospitalization due to all causes, infectious diseases, and respiratory infections. For every outcome, hazard ratios (HR) with 95% confidence interval (CI) were derived from included trials for vaccinated compared to unvaccinated participants by using direct and indirect methods according to Tierney et al. [21]. For meta-analysis, a random effects model was assumed. As sensitivity analysis, we used a fixed effect model. We pooled derived HRs by using the generic inverse variance method. We assessed heterogeneity using Chi^2^-tests and quantified heterogeneity using the I^2^ statistic; a *p*-value < 0.05 was considered significant [22]. For every outcome with at least nine studies, we evaluated potential bias by visual inspection of funnel plot asymmetry [23]; we assessed potential causes of heterogeneity by stratifying the analysis by age, health status, trial region, method of outcome collection, and follow-up time. Contribution of individual trials to the overall result was analyzed by excluding one study at a time. Review Manager (RevMan, version 5.3.5, 2018) was used for all the analyses.

## 3. Results

### 3.1. Identification of Studies

**Figure 1:**
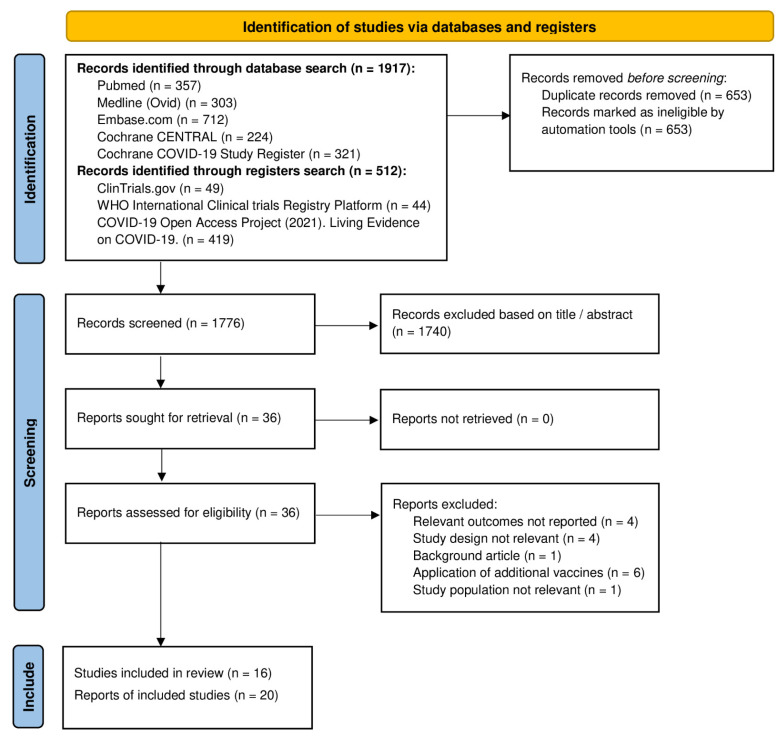
Identification and selection of eligible trials for inclusion in meta-analysis.

The literature search identified 2429 records. 1917 records were from databases and 512 records from registers. After removal of 653 duplicates, 1776 potentially relevant references and citations describing NSE after BCG vaccination were identified and screened for retrieval. Of these, we excluded 1740 reports based on title and abstract because they did not meet the inclusion criteria. The remaining 36 articles were selected for full-text analysis and evaluated in more detail. Of these, 16 were excluded for the following reasons: four articles did not report relevant outcomes, four articles described trials with a study design that was not relevant, one article was a background article, in one trial the study population was not relevant [24], and in six trials participants received additional vaccines during the follow-up period [25,26,27,28,29,30]. The remaining 20 articles reported about 16 trials which met all the inclusion- and exclusion criteria and were included in the systematic review and meta-analysis (Figure 1) [31].

### 3.2. Characteristics of Included Studies

The 16 identified trials were done in twelve different countries representing low- and high income settings: Guinea-Bissau, Malawi, Uganda, South Africa, Brazil, Indonesia, India, Australia, Denmark, the Netherlands, Greece, and Germany (Table 1). 

The trials included a total of 34,197 participants. The median number of participants per study was 1272 (interquartile range (IQR) 3072-265). Seven trials (44%) were conducted in newborn children [4,5,6,7,8,32,33], one (6%) in adolescents [34], three (19%) in adults [35,36,37], and four trials (25%) in elderly [16,38,39,40]. One trial (6%) included all age groups [41]. Eight trials (50%) mainly included Black participants [4,5,6,33,34,35,37,41], two trials (13%) mainly Asian participants [32,38], and six trials (38%) were conducted with participants of mainly Caucasian ethnicity [7,8,16,36,39,40]. Ten trials (63%) were conducted in healthy individuals [7,8,33,34,35,36,37,38,40,41], two (13%) included participants with various comorbidities [16,39], and four (25%) were done in low-birth-weight children (<2500 g) [4,5,6,32]. The primary objectives of 15 trials (94%) was to evaluate BCG-mediated NSE on mortality, infections of any origin, and respiratory infections [4,5,6,7,8,16,32,33,35,36,37,38,39,40,41]; five trials (31%) specifically investigated BCG-mediated NSE on COVID-19 [16,35,36,37,40]; one (6%) trial was conducted to study prevention of Mycobacterium tuberculosis infection with BCG revaccination [34]. As intervention the BCG Denmark strain was used in ten trials (63%) [4,5,6,7,8,33,34,36,37,39]; one trial (6%) used the BCG Glaxo-strain which is genetically close to the BCG Denmark strain [41]; two trials (13%) used the BCG Moscow strain [16,37]; one trial (6%) used the strain VPM1002 [40]; the remaining two trials (13%) used the strains BCG Paris or BCG Russia, respectively [32,38]. Participants were reported to be naïve for BCG in seven trials (44%) [4,5,6,7,8,32,33]. Seven trials (44%) evaluated revaccination with BCG [34,35,36,37,38,40,41], one of these trials applied BCG once a month for three months in succession [38]. Two (13%) trials did not report previous BCG vaccination status [16,39]. As control intervention placebo was used in eight trials (50%) [16,34,36,37,38,39,40,41]; three trials (19%) used no intervention as control [7,8,35]; five trials (31%) applied in the control group BCG later according to local policies [4,5,6,32,33]. The follow-up period was in thirteen trials (81%) shorter or equal to one year [4,5,6,8,16,32,33,35,36,37,38,39,40]; two trials (13%) had a follow-up between 12 and 24 months [7,34]; one trial (6%) had follow-up data for four years in one population and for 16 years in another population [41]. Information about the application of additional vaccines after BCG within the follow-up period was provided in seven trials (44%) [7,8,35,36,37,40,41]. 

### 3.3. Study Quality

Risk of bias assessment of included trials is shown in Table 2. Two trials (13%) did not report the method of randomization [16,38], and six trials (38%) did not describe the method of allocation concealment [6,8,16,36,38,41]. Blinding of participants and personnel was unclear in one trial (6%) [38] and was judged to have a high risk of bias in seven trials (44%) [8,16,35,36,37,40,42], in particular, due to the collection of participant-reported outcomes without medical diagnosis and visible scar formation after BCG vaccination. For the objective outcome mortality, blinding of participants and personnel was judged to have a low risk of bias. In one trial (6%) blinding of assessment was unclear [38], in another trial (6%) it was judged to cause a high risk of bias [36]. Incomplete outcome data due to attrition bias was judged to cause a high risk of bias in six trials (38%) [16,35,36,37,40,41]. 15 trials (94%) included an intention-to-treat analysis [4,5,6,8,16,32,33,34,35,36,37,39,40,41,42]. One trial (6%) did not inform about the type of analysis [38].

### 3.4. Meta-Analysis

#### 3.4.1. Effects of BCG on Non-Tuberculosis Respiratory Infections and COVID-19

We identified nine RCTs including 8062 participants reporting NSE of BCG on non-tuberculosis respiratory infections (Table 3). 

**Figure 2:**
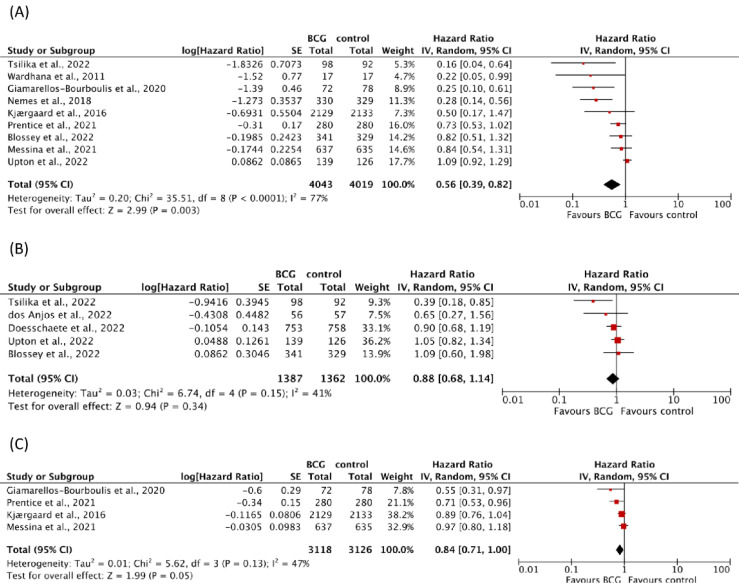
Forest plots of random-effects meta-analysis of BCG trials for (**A**) respiratory infections, (**B**) COVID-19, and (**C**) infections of any origin. Solid squares represent hazard ratio estimates for the single studies. The size of the squares represents the weight assigned to the individual study in the meta-analysis and is proportional to the inverse variance (IV) of the estimate. Horizontal lines indicate 95% confidence intervals (CI). The diamond shows the 95% CI for the pooled hazard ratios. Values smaller than 1.0 indicate hazard ratios that favor BCG. BCG = Bacillus Calmette-Guérin, SE = standard error.

Three were conducted in healthy newborns (weight 37.9%) [7,8,33], one in adolescents who already received BCG in infancy (weight 16%) [34], one in adults (weight 17.7%) [37], and four in elderly with unknown previous BCG exposure (weight 33.1%) [16,38,39,40]. Combined HRs from random-effects meta-analyses indicated a beneficial effect of the vaccine on non-tuberculosis respiratory infections (HR 0.56, 95% CI 0.39–0.82); heterogeneity between trials was substantial (I^2^ = 77%; *p* < 0.0001) (Figure 2A). 

Visual inspection of funnel plots revealed a tendency of small trials to lead to more beneficial intervention effect estimates (Figure S1). Regarding the risk of diagnosed COVID-19 in COVID-19 naïve individuals, five RCTs including 2749 participants indicated no evidence for a protective effect after BCG vaccination (HR 0.88, 95% CI 0.68–1.14) with moderate heterogeneity between trials (I^2^ = 41%; *p* = 0.15) (Figure 2B). Two of them included elderly participants (weight 23.2%) [16,40] and three were conducted in adult healthcare workers (weight 76.8%) [35,36,37]. Results remained similar when we used a fixed effect model or excluded one study at a time.

#### 3.4.2. Effects of BCG on Infections of Any Origin and Sepsis

Four RCTs with a total of 6244 participants investigated the effect of BCG on infections of any origin (Table 3). Three trials were conducted in BCG-naïve healthy newborns (weight 92.2%) [7,8,33], and one trial was conducted in elderly (weight 7.8%) [39]. There was no evidence for a protective BCG-mediated effect (HR 0.84, 95% CI 0.71–1.00) with moderate heterogeneity between trials (I^2^ = 47%; *p* = 0.13) (Figure 2C). Exclusion of one study at a time did not extensively change the overall effect. However, applying a fixed effect model resulted in a statistically significant protective effect (HR 0.87, 95% CI 0.78–0.97) (Figure S2).

Regarding sepsis, three RCTs with 7293 participants indicated no evidence for a protective NSE of BCG (HR 0.78, 95% CI 0.55–1.10; I^2^ = 0%; *p* = 0.97) (Table 3) [33,39,43].

#### 3.4.3. Effects of BCG on Mortality

**Figure 3:**
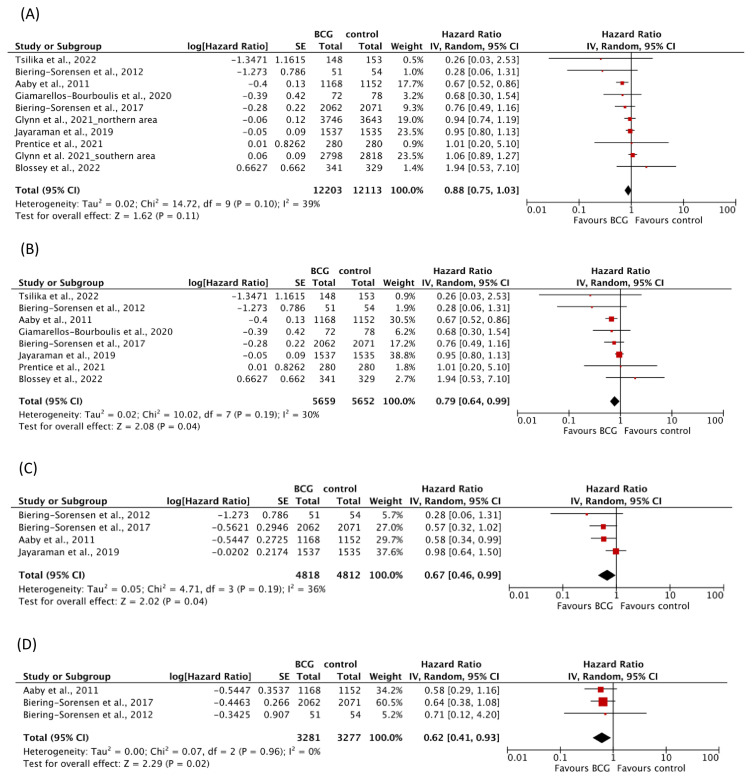
Forest plots of random-effects meta-analysis of BCG trials for (**A**) all-cause mortality, (**B**) all-cause mortality with one year follow-up, (**C**) mortality for infections, and (**D**) mortality for sepsis. Solid squares represent hazard ratio estimates for the single studies. The size of the squares represents the weight assigned to the individual study in the meta-analysis and is proportional to the inverse variance (IV) of the estimate. Horizontal lines indicate 95% confidence intervals (CI). The diamond shows the 95% CI for the pooled hazard ratios. Values smaller than 1.0 indicate hazard ratios that favor BCG. BCG = Bacillus Calmette-Guérin, SE = standard error.

Nine RCTs with a total of 24,316 participants analyzed BCG-mediated effects on all-cause mortality (Table 3). One study followed two populations in two different areas (northern and southern areas) during different time periods with different methods of follow-up and therefore contributed two HRs [41]. There was no evidence for an effect of BCG vaccination on all-cause mortality (HR 0.88, 95% CI 0.75–1.03) with moderate heterogeneity between the trials (I^2^ = 39%; *p* = 0.10) (Figure 3A). Restriction to trials with a follow-up of one year excluded one trial [41] which contributed 42.5% weight to the overall analysis and resulted in a statistically significant protective effect (HR 0.79, 95% CI 0.64–0.99) and reduced in-between trial heterogeneity (I^2^ = 30%; *p* = 0.19) (Figure 3B). 

Evidence from four trials indicated a protective effect of BCG on infection-related mortality (HR 0.67, 95% CI 0.46–0.99) with moderate heterogeneity between trials (I^2^ = 36%; *p* = 0.19) (Figure 3C).

Three trials reported results about mortality due to respiratory infections, indicating no evidence for an effect (HR 0.47, 95% CI 0.18–1.24) (I^2^ = 0%; *p* = 0.84) (Table 3) [4,6].

Mortality for sepsis after BCG vaccination was reported in three trials and resulted in a significantly reduced overall effect estimate (HR 0.62, 95% CI 0.41–0.93) (I^2^ = 0%; *p* = 0.96) (Figure 3D). Results with fixed effect and random effects models were similar.

#### 3.4.4. Effects of BCG on Hospitalization

We identified nine RCTs including 13,367 participants investigating effects of BCG vaccination on all-cause hospitalization (Table 3). The combined HR was 1.01 (0.91–1.11) indicating no evidence for an effect. Trial results were homogenous (I^2^ = 0%; *p* = 0.70) and the results with fixed effect and random effects models were identical (Figure S3). Meta-analysis of three trials investigating infectious disease hospitalization and of four trials examining hospitalization for respiratory infections showed no statistically significant improvement after BCG vaccination with combined hazard ratios of 0.96 (95% CI 0.85–1.10) and 0.64 (95% CI 0.27–1.53), respectively (Figure S3). We found no additional RCTs reporting about hospitalization due to sepsis apart from three RCTs which where meta-analyzed by others before [43].

#### 3.4.5. Subgroup Analysis

Meta-analysis for the outcomes non-tuberculosis respiratory infections (nine trials), COVID-19 (five trials), infections of any origin (four trials), and mortality (nine trials) showed moderate to substantial heterogeneity between the trials (Table 3). We performed subgroup analyses for outcomes with at least nine trials by stratifying for age, health status, trial region, follow-up period, and method of outcome collection (Table 4). For non-tuberculosis respiratory infections, there was evidence for statistically significant differences between all subgroups (test for subgroup differences: I^2^ = 77%; *p* < 0.0001), suggesting more pronounced BCG-mediated NSE in non-tuberculosis respiratory infections (1) in adolescents or adults, (2) in low-birth-weight children or morbid participants, (3) in trials conducted in Western Europe or Australia, (4) in trials with a follow-up period smaller or equal to six months, (5) and in trials collecting outcome data by medical diagnosis compared to participant-reported data. 

For all-cause mortality, there was evidence for a statistically significant difference between subgroups with different health status, suggesting more pronounced BCG-mediated NSE on mortality in low birth-weight children or morbid participants compared to others (Table 4).

## 4. Discussion

Our meta-analysis indicates that vaccination with BCG caused an estimated 44% decrease in risk for non-tuberculosis respiratory infections (HR 0.56, 95% CI 0.39–0.82). Additional analyses revealed evidence for a protective effect after BCG vaccination of 21% on all-cause mortality if follow-up was restricted to one year (HR 0.79, 95% CI 0.64–0.99); there was no evidence for an effect when longer follow-up was considered (HR 0.88, 95% CI 0.75–1.03). In particular, mortality for infections (HR 0.67, 95% CI 0.46–0.99) and mortality for sepsis (HR 0.62, 95% CI 0.41–0.93) were significantly reduced after BCG vaccination. For COVID-19 (HR 0.88, 95% CI 0.68–1.14), infections of any origin (HR 0.84, 95% CI 0.71–1.00), and sepsis (HR 0.78, 95% CI 0.55–1.10), there was no evidence for a protection after BCG vaccination. Regarding hospitalization (HR 1.01, 95% CI 0.91–1.11), hospitalization for infections (HR 0.96, 95% CI 0.85–1.10), and hospitalization for respiratory infections (HR 0.64, 95% CI 0.27–1.53), we found no evidence for improvement after BCG vaccination. Subgroup analyses suggested better protection of non-tuberculosis respiratory infections after BCG vaccination in adolescents or adults as compared to infants; in participants with impaired health including low-birth-weight children as compared to others; in trials from Western Europe or Australia as compared to trials from Africa, Indonesia, India, South America; in trials with a follow-up period shorter or equal to six months; and in trials with outcome collection by a medical diagnosis instead of participant-reported data. Regarding all-cause mortality, subgroup analyses indicated better protection in low-birth-weight children or morbid participants as compared to others.

Based on the few RCTs included and the large number of potential effect modifiers and confounders, results of our subgroup-analysis must be interpreted with caution. The trials indicating less pronounced NSE for respiratory infections in infants include only three RCTs [8,33,44]; two of them collected outcome data by interviewing parents of participating infants [8,42]. Diagnosing a respiratory infection in infants under 13 months of age might be challenging for a lay-person without medical education, in particular as these two RCTs were originally designed to study the effect of BCG on allergies and therefore included a study population with a high degree of families with a history of atopy and asthma [45]. Therefore, allergic predisposition among infants might have influenced this outcome. Moreover, BCG vaccination causes a visible scar hindering the blinding of participants and thereby introducing a possible source of bias. Results of our stratification by the method of outcome collection support this assumption and suggest more pronounced BCG-mediated protective effects in trials with data collection based on a medical diagnosis. However, subgroup analysis of mortality, an objective outcome that is not affected by the method of outcome collection, indicates more pronounced protective effects in infants compared to adults. 

The effect of routine childhood vaccinations on overall mortality was first systematically analyzed in observational studies in Guinea-Bissau. A large cohort study revealed that BCG vaccination was associated with a significantly lower mortality ratio of 0.55 (95% CI 0.36–0.85) [46]. These promising results led to a systematic review commissioned by the WHO which identified five trials with an average relative risk of 0.70 (95% CI 0.49–1.01) and nine observational studies with an average relative risk of 0.47 (95% CI 0.32–0.69) in 2013. Mortality reduction was most significant in two trials that were restricted to infants with low-birth-weight (RR 0.52, 95% CI 0.33–0.82) [3]. Another meta-analysis of three RCTs conducted in Guinea-Bissau showed that early BCG administration reduced mortality by 38% within the neonatal period (MRR 0.62, 95% CI 0.46–0.83) [6]. In 2014, the strategic advisory group of experts (SAGE) demanded a further confirmation of the results about NSE via the conduction of high quality RCTs [47]. In line with this recommendation, several research groups worldwide published additional RCTs investigating non-specific effects of BCG, but with different results. Importantly, the majority of the by SAGE demanded high quality RCTs were published after 2013 and therefore not included in the work by Higgins et al., 2016. According to our information, there was no systematic search and meta-analysis of relevant clinical outcomes from RCTs published since then.

Our meta-analysis is based on data from 16 RCTs reporting NSE of BCG; 13 of them were published after 2017 and, to our knowledge, not included in previous meta-analyses, six of these 13 report mortality data. Based on this evidence, we did not find a significant reduction in all-cause mortality after BCG vaccination (HR 0.88, 95% CI 0.75–1.03). However, restriction to a follow-up of twelve months excluded one RCT [41], which contributed 43% weight to the overall analysis, and resulted in an overall significant protective effect in a range of previous findings (HR 0.79, 95% CI 0.64–0.99). This large RCT in Malawi [41] followed two populations for four and 16 years but could not detect any protective NSE after BCG vaccination. The authors concluded that the large number of non-infectious related deaths and the long time interval since BCG vaccination might have obscured any protective NSE. 

The persistence of NSE of BCG has been analyzed in mechanistic studies demonstrating epigenetic reprogramming of monocytes, leading to increased cytokine production in response to non-related pathogens for up to three months after BCG vaccination, and enduring changes in pattern recognition receptors after one year. In healthy humans, non-specific Th1 and Th17 responses were enhanced for at least one year after vaccination [10]. In addition to this trained immunity, emergency granulopoiesis has recently been described as a mechanism providing non-specific protection within days of BCG-administration [12]. Moreover, heterologous T cell reactivity may also play a role [13]. Nevertheless, it is still unclear how long NSE after BCG vaccination last and whether they can be reversed. 

Whereas the previous meta-analysis quantified the impact of NSE of BCG on all-cause mortality in children, we focused our meta-analysis on non-tuberculosis respiratory infections and COVID-19 and considered additional age groups. Approximately 40 ongoing RCTs on this subject are a unique chance to elucidate the question of whether, and under which circumstances, BCG protects from COVID-19 [15]. However, the launch of national COVID-19 vaccination programs interfered with ongoing BCG-COVID-19 trials. COVID-19-vaccinated people do not qualify for studies examining NSE of BCG alone on COVID-19. By the end of our search, only five RCTs reporting data from participants not vaccinated with a COVID-19-specific vaccine were published and could be included in our analysis [16,35,36,37,40]. The number of people who are not willing to get vaccinated with a COVID-19 vaccine but volunteer for a BCG-COVID-19 trial is probably limited which impedes the recruitment of study participants. Due to this fact, it is questionable as to how many of these BCG-COVID-19 trials will be finished and when this data will be available. Our meta-analysis of published BCG-COVID-19 trials does not provide evidence for a protective effect of BCG vaccination on COVID-19. However, a recent RCT conducted in adults with type 1 diabetes demonstrated a protective effect of multiple BCG vaccinations on COVID-19 with a vaccine efficacy of 92%. Moreover, BCG-vaccinated patients exhibited fewer infections and fewer infectious disease symptoms and severity [48]. Although we excluded this trial from our review because of study participants with severe comorbidities, it supports the findings of our subgroup analysis indicating increased BCG-mediated NSE for respiratory infections in participants with impaired health. 

Several effective COVID-19 specific vaccines exist by now. Nevertheless, the question of whether BCG can protect from respiratory infections in general is still highly relevant. (1) BCG’s ability to train the immune system and thereby protect antigen-independent against infections might provide an effective shield against vaccine-resistant mutants, which are able to circumvent antigen-specific immunological memory. Thus, BCG might be valuable to bridge time until an antigen-specific vaccine against a dangerous vaccine-resistant mutant is developed [49]. (2) BCG is safe, relatively inexpensive, and can easily be provided on a mass scale [50]. According to current WHO policy, BCG is used for neonates in vaccination programs in developing countries in which the access to specific vaccines and medical supply is usually impaired [2]. This wide availability of BCG might be of strategic importance in pandemic preparedness: it could be easily provided and quickly be used for BCG-revaccination programs—with the potential to short-term prevent viral spread in these countries and therefore become a game-changer in the course of a future pandemic. (3) Antimicrobial resistance is a global health and development threat. It was declared by the WHO as one of the top 10 global public health threats facing humanity [51]. Globally, drug-resistant infections caused due to antimicrobial resistance contribute to about 700,000 deaths annually. Without effective intervention, drug-resistant infections are projected to cause 10 million deaths and a global economic loss of USD 100 trillion by 2050 [52]. BCG-mediated training of the immune system might not only reduce the burden of infectious diseases, but also decrease the need for antimicrobial prescriptions, thereby lowering the risk for the development of further drug-resistant pathogens.

## 5. Conclusions

With rising resistance to antimicrobial interventions worldwide, improving the host response to infections becomes increasingly important. This meta-analysis summarizes the recent available RCT-data on BCG-mediated NSE and indicates time-limited and partial protection from respiratory infections. There was no evidence for a protective NSE of BCG vaccination on COVID-19. Yet, the BCG-mediated NSE described here have the potential to short-term prevent viral spread and reduce pandemic-related mortality, thus being of strategic importance in pandemic preparedness. Especially in developing countries with established BCG vaccination programs, BCG is easily accessible for revaccinations in pandemic emergencies which could positively influence the course of a future pandemic. Moreover, BCG-mediated NSE reduce microbial infections in general, thereby decreasing antimicrobial prescriptions and thus the development of antimicrobial resistance.

Our findings indicated a protective NSE on mortality within one year after BCG vaccination and support the current WHO policy of providing BCG vaccination to all infants on the first day of life in areas of high infectious disease incidence [2]. However, as the setup of the included RCTs is heterogeneous, the number of potential confounders and effect modifiers is large. There is a need for additional RCTs to clarify the circumstances under which BCG mediates NSE.

## Figures and Tables

**Table 1:** Characteristics of randomized controlled trials investigating non-specific effects of the BCG vaccine included in meta-analysis. BCG = Bacillus Calmette-Guérin.

Lead Author, Publication Date	Location	Study Period	Age Group	Intervention (BCG Type)	Time of Intervention	Control	Follow-Up	Total Trial Participants	Health Status
Aaby et al., 2011 Schaltz-Buchholzer et al., 2013 Schaltz-Buchholzer et al., 2018	Guinea-Bissau	Nov 2002–Mar 2008	newborn children	BCG Denmark	immediately after birth	BCG later, i.e., when the child had gained weight or at 6 weeks of age	12 months	2320	low birth weight children < 2500 g
Wardhana et al., 2011	Indonesia	Jun 2009–Nov 2009	age 60–75 years	BCG weakened living Mycobacterium bovis Pasteur Paris strain mp 1173-P2 produced by PT Biofarma, Bandung, Indonesia	once a month for 3 months in succession	Placebo	6 months	34	healthy individuals
Biering-Sorensen et al., 2012 Schaltz-Buchholzer et al., 2018	Guinea-Bissau	Nov 2004–Mar 2008	newborn children	BCG Denmark	immediately after birth	BCG later, i.e., when the child had gained weight or at 6 weeks of age	12 months	104	low birth weight children < 2500 g
Kjærgaard et al., 2016 Stensballe et al., 2017 Stensballe et al., 2019	Denmark	Sep 2012–Jan 2015	newborn children	BCG Denmark	within 7 days of age	no intervention	13 months	4262	healthy newborns
Biering-Sorensen et al., 2017 Schaltz-Buchholzer et al., 2018	Guinea-Bissau	Feb 2008–Sep 2013	newborn children	BCG Denmark	immediately after birth	BCG later, i.e., when the child had gained weight or at 6 weeks of age	12 months	4154	low birth weight children < 2500 g
Nemes et al., 2018	South Africa	May 2015–Dec 2016	age 12–17 years	BCG Denmark	revaccination on day 0 (participants were BCG vaccinated in infancy)	Placebo	24 months	989	healthy individuals
Jayaraman et al., 2019	India	Oct 2013–not reported	newborn children	BCG-Russia	immediately after birth	BCG later, i.e., 28 days after birth	28 days	3072	low birth weight children < 2000 g
Giamarellos-Bourboulis et al., 2020	Greece	Sep 2017–Nov 2020	age ≥ 65 years	BCG Denmark	Day of hospital discharge	Placebo	12 months	198	Elderly participants discharged from hospital with various comorbidities
Glynn et al., 2021	Karonga District, northern Malawi	Enrollment of partcipants: Jan 1986–Nov 1989	3 months–75 years	BCG (Glaxo-strain) revaccination	revaccination after randomisation	Placebo	Northern area: 1991–1994 by active follow-up, Southern area: 2002–2018 by demographic surveillance	Northern area: 7389, Southern area: 5616	Excluded were individuals with past or current leprosy or tuberculosis, severe malnutrition, or other severe illness.
Messina et al., 2021	Australia	Aug 2013–Sep 2016	newborn children	BCG Denmark	first 10 days of life	no intervention	12 months	1272	healthy newborns
Prentice et al., 2021	Uganda	Mar 2014–Jul 2015	newborn children	BCG Denmark	immediately after birth	BCG later, i.e., 6 weeks after birth	6 weeks	560	healthy infants
Tsilika et al. 2022	Greece	May 2020–May 2021	age ≥ 50 years; mean age 69 years	BCG Moscow	day of hospital discharge	Placebo	6 months	301	Elderly participants discharged from hospital with various comorbidities
dos Anjos et al., 2022	Brazil	Aug 2020–Aug 2021	adult healthcare workers	BCG Moscow	revaccination after randomisation	no intervention	180 days	113	healthy individuals
Doesschate et al., 2022	Netherlands	Mar 2020–Apr 2021	adult healthcare workers	BCG Denmark	(re-)vaccination after randomisation	Placebo	1 year	1511	healthy individuals
Upton et al., 2022	South Africa	Enrollment of participants: May 2020–Oct 2020	adult healthcare workers	BCG Denmark	revaccination after randomisation	Placebo	52 weeks	265 (per-protocol analysis)	healthy individuals (48.5% with latent tuberculosis)
Blossey et al., 2022	Germany	Jun 2020–Oct 2021	age ≥ 60 years	VPM1002	(re-)vaccination after randomisation	Placebo	240 days	2037	healthy individuals

**Table 2:** Risk of bias in RCTs. White/Grey: low risk of bias; pattern ////////: unclear risk of bias; black: high risk of bias.

Author Name, Year	Random Sequence Generation	Allocation Concealment	Blinding of Participants and Personnel	Blinding of Assessment	Incomplete Outcome Data	Selective Reporting	Other Bias *
Aaby et al., 2011 Schaltz-Buchholzer et al., 2013 Schaltz-Buchholzer et al., 2018							
Wardhana et al., 2011	/////////////////	/////////////////	/////////////////	/////////////////			
Biering-Sorensen et al., 2012 Schaltz-Buchholzer et al., 2018							
Kjærgaard et al., 2016 Stensballe et al., 2017 Stensballe et al., 2019							//////////////
Biering-Sorensen et al., 2017 Schaltz-Buchholzer et al., 2018		/////////////////					//////////////
Nemes et al., 2018							
Jayaraman et al., 2019							
Giamarellos-Bourboulis et al., 2020							//////////////
Glynn et al., 2021		/////////////////					//////////////
Messina et al., 2021		/////////////////					//////////////
Prentice et al., 2021							
Tsilika et al. 2022	/////////////////	/////////////////					//////////////
Dos Anjos et al. 2022							//////////////
Doesschaete et al. 2022		/////////////////					//////////////
Upton et al. 2022							//////////////
Blossey et al., 2022							//////////////

* Other bias refers to bias due to problems not covered elsewhere in the table (e.g., the study had a potential source of bias related to the specific study design used; or there is insufficient information to assess whether an important risk of bias exists; or insufficient rationale or evidence that an identified problem will introduce bias).

**Table 3:** Hazard ratios of respiratory infections, COVID-19, infections of any origin, sepsis, mortality, and hospitalization, according to random-effects meta-analysis of included randomized controlled trials of BCG vaccine that report these outcomes. HR = hazard ratio, CI = confidence interval.

Outcome	No. of Trials	No. of Study Participants	No. of Cases	Combined HR (95% CI)	Test for Heterogeneity
Respiratory infections	9	8062	902	0.56 (0.39–0.82)	I^2^ = 77%; *p* < 0.0001
COVID-19	5	2749	263	0.88 (0.68–1.14)	I^2^ = 41%; *p* = 0.15
Infections of any origin	4	6244	1298	0.84 (0.71–1.00)	I^2^ = 47%; *p* = 0.13
Sepsis	3	7293	117	0.78 (0.55–1.10)	I^2^ = 0%; *p* = 0.97
Mortality	9	24,316	1452	0.88 (0.75–1.03)	I^2^ = 39%; *p* = 0.10
Mortality, follow-up ≤ 1 year	8	11,311	936	0.79 (0.64–0.99)	I^2^ = 30%; *p* = 0.19
Mortality for infections	4	9630	194	0.67 (0.46–0.99)	I^2^ = 36%; *p* = 0.19
Mortality for respiratory infections	3	7123	16	0.47 (0.18–1.24)	I^2^ = 0%; *p* = 0.84
Mortality for sepsis	3	6558	94	0.62 (0.41–0.93)	I^2^ = 0%; *p* = 0.96
Hospitalization	9	13,367	2516	1.01 (0.91–1.11)	I^2^ = 0%; *p* = 0.70
Hospitalization for infections	3	12,117	886	0.96 (0.85–1.10)	I^2^ = 0%; *p* = 0.72
Hospitalization for respiratory infections	4	7708	45	0.64 (0.27–1.53)	I^2^ = 52%; *p* = 0.10

**Table 4:** Subgroup analysis of BCG-trials for the outcomes respiratory infections and all-cause mortality. Outcomes were stratified by age, health status, trial region, follow-up period, and method of outcome collection. For trials conducted in infants and for COVID-19 related trials a follow-up period before the application of additional vaccines was used. HR = hazard ratio, CI = confidence interval.

	Respiratory Infections						All-Cause Mortality					
			Subgroup Heterogeneity		Test for Subgroup Differences				Subgroup Heterogeneity		Test for Subgroup Differences	
Variable	No. of Trials	HR [95% CI]	I^2^ [%]	*p*-Value	I^2^ [%]	*p*-Value	No. of Trials	HR [95% CI]	I^2^ [%]	*p*-Value	I^2^ [%]	*p*-Value
**Age**												
infants	3	0.75 [0.58, 0.97]	0	0.67	77	<0.0001	5	0.79 [0.62, 1.00]	44	0.13	39	0.1
adolescents or adults	6	0.43 [0.22, 0.83]	85	<0.00001			4	1.01 [0.88, 1.16]	0	0.42		
**Health status**												
low birth-weight children or morbid participants	2	0.22 [0.10, 0.46]	0	0.6	77	<0.0001	6	0.81 [0.71, 0.92]	52	0.06	49	0.04
other	7	0.69 [0.49, 0.97]	73	0.001			3	1.02 [0.89, 1.18]	0	0.66		
**Trial region**												
Western Europe, Australia	5	0.52 [0.30, 0.89]	63	0.03	77	<0.0001	3	0.84 [0.34, 2.04]	31	0.24	39	0.1
Africa, Indonesia, India, South America	4	0.59 [0.33, 1.05]	85	0.0001			6	0.88 [0.75, 1.04]	49	0.07		
**Follow-up period**												
≤6 months	3	0.35 [0.12, 1.07]	69	0.04	77	<0.0001	6	0.78 [0.61, 0.99]	39	0.15	39	0.1
>6 months	6	0.61 [0.39, 0.97]	80	0.0002			3	1.01 [0.88, 1.16]	0	0.47		
**Method of outcome collection**												
participant reported outcome without medical diagnosis	5	0.79 [0.54, 1.15]	62	0.03	77	<0.0001	0	-	-	-	-	-
medical diagnosed outcome	4	0.37 [0.18, 0.75]	72	0.01			9	0.88 [0.75, 1.03]	39	0.1		

## Data Availability

The authors confirm that the data supporting the findings of this study are available within the article and/or its Supplementary Materials.

## Supplementary Materials

The following supporting information can be downloaded at: https://www.mdpi.com/article/10.3390/vaccines11010121/s1. Figure S1: Funnel plots of random-effects meta-analysis of BCG trials for outcomes with at least nine studies; Figure S2: Forest plot of fixed-effect meta-analysis of BCG trials for infections of any origin; Figure S3: Forest plots of random-effects meta-analysis of BCG trials for (A) hospitalization, (B) hospitalization for infections, and (C) hospitalization for respiratory infections; Table S1: Electronic data sources for systematic literature search; Table S2: Detailed search strategy used in this review; Table S3: Review checklist with in- and exclusion criteria.
