# Supplementary material for: Non-Specific Effects of Bacillus Calmette-Guérin: A Systematic Review and Meta-Analysis of Randomized Controlled Trials
Source: Vaccines (Basel). 2023 Jan 4;11(1):121. doi: 10.3390/vaccines11010121 (PMC9866113; doi:10.3390/vaccines11010121)
Supplement: Supplementary file 1 [file vaccines-11-00121-s001.zip › vaccines-2097950-Supplementary Figures.pdf]

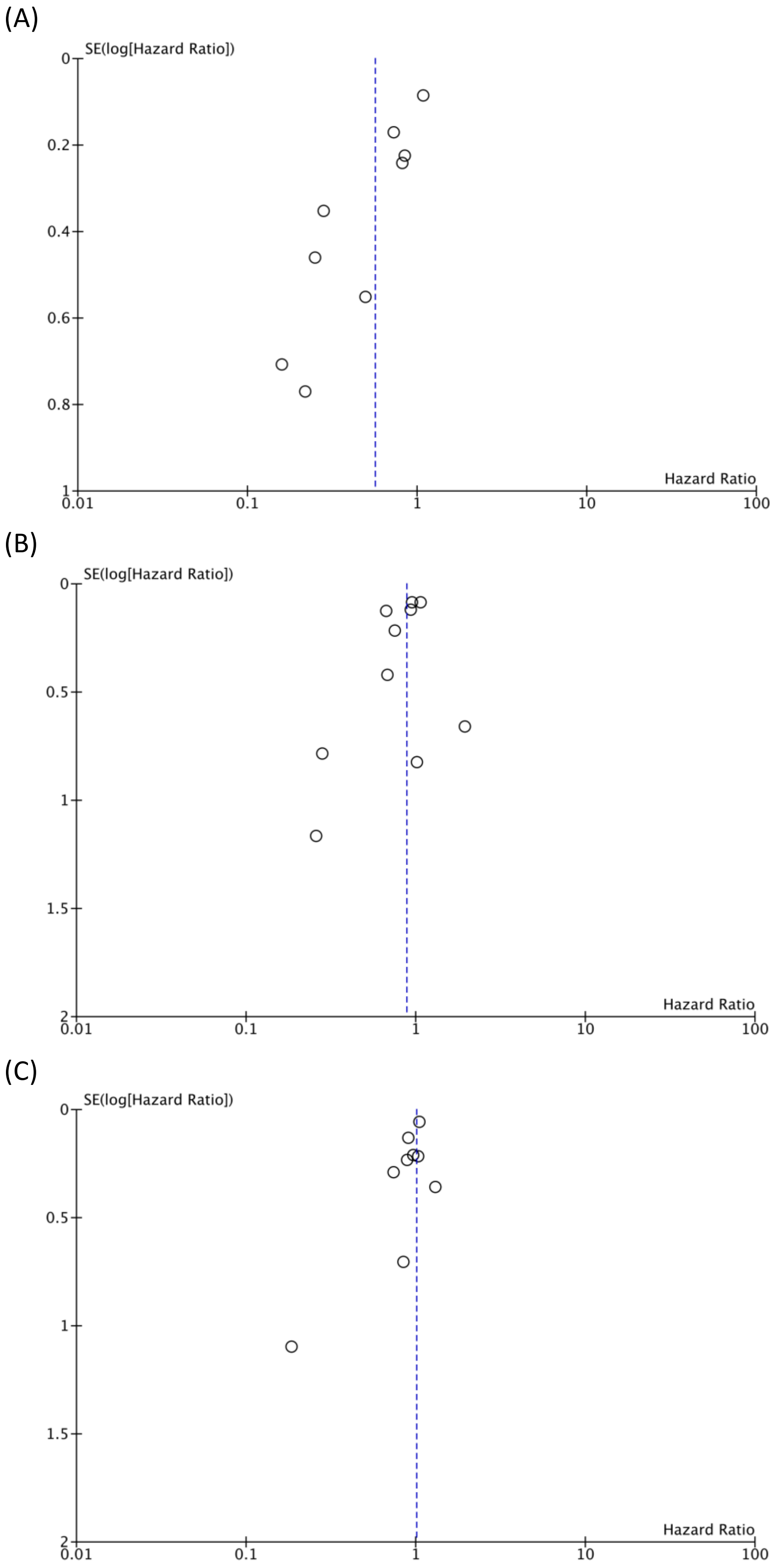

Figure S1. Funnel plots of random-effects meta-analysis of BCG trials for outcomes with at least nine studies. (A) Respiratory infections (B) All-cause mortality (C) All-cause hospitalization. SE = standard error

Study or Subgroup	log[Hazard Ratio]	SE	BCG Total	control Total	Weight	Hazard Ratio IV, Fixed, 95% CI
Giamarellos–Bourboulis et al., 2020	−0.6	0.29	72	78	3.8%	0.55 [0.31, 0.97]
Prentice et al., 2021	−0.34	0.15	280	280	14.2%	0.71 [0.53, 0.96]
Kjærgaard et al., 2016	−0.1165	0.0806	2129	2133	49.1%	0.89 [0.76, 1.04]
Messina et al., 2021	−0.0305	0.0983	637	635	33.0%	0.97 [0.80, 1.18]
**Total (95% CI)**			**3118**	**3126**	**100.0%**	**0.87 [0.78, 0.97]**

Heterogeneity: $Chi^2 = 5.62$, df = 3 (P = 0.13); $I^2 = 47\%$

Test for overall effect: Z = 2.45 (P = 0.01)

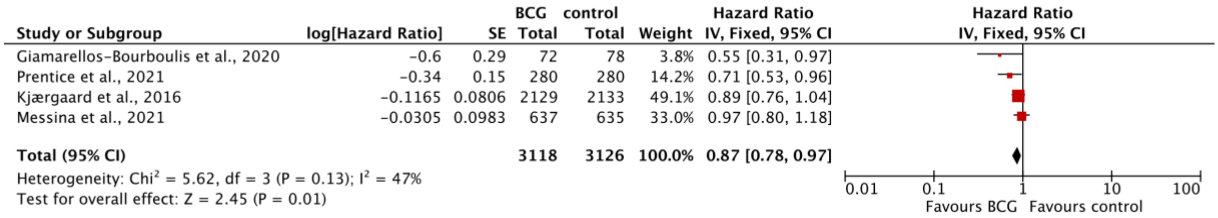

Figure S2. Forest plot of fixed-effect meta-analysis of BCG trials for infections of any origin. Solid squares represent hazard ratio estimates for the single studies. The size of the squares represents the weight assigned to the individual study in the meta-analysis and is proportional to the inverse variance (IV) of the estimate. Horizontal lines indicate 95% confidence intervals (CI). The diamond shows the 95% CI for the pooled hazard ratios. Values smaller than 1.0 indicate hazard ratios that favour BCG. BCG = Bacillus Calmette-Guérin, SE = standard error

(A)

Study or Subgroup	log[Hazard Ratio]	SE	BCG Total	control Total	Weight	Hazard Ratio IV, Random, 95% CI
Tsilika et al., 2022	−1.6901	1.095	98	92	0.2%	0.18 [0.02, 1.58]
Giamarellos-Bourboulis et al., 2020	−0.3011	0.289	72	78	3.0%	0.74 [0.42, 1.30]
Biering-Sorensen et al., 2012	−0.1744	0.7073	51	54	0.5%	0.84 [0.21, 3.36]
Schaltz-Buchholzer et al., 2013	−0.1165	0.2364	1168	1152	4.5%	0.89 [0.56, 1.41]
Blossey et al., 2022	−0.0943	0.1339	341	329	13.9%	0.91 [0.70, 1.18]
Messina et al., 2021	−0.03	0.21	637	635	5.7%	0.97 [0.64, 1.46]
Biering-Sorensen et al., 2017	0.0392	0.2168	2062	2071	5.3%	1.04 [0.68, 1.59]
Stensballe et al., 2017	0.0488	0.0619	2129	2133	65.0%	1.05 [0.93, 1.19]
Upton et al., 2022	0.27	0.3576	139	126	1.9%	1.31 [0.65, 2.64]
**Total (95% CI)**			**6697**	**6670**	**100.0%**	**1.01 [0.91, 1.11]**

Heterogeneity: $Tau^2 = 0.00$; $Chi^2 = 5.50$, df = 8 (P = 0.70); $I^2 = 0\%$
Test for overall effect: Z = 0.11 (P = 0.91)

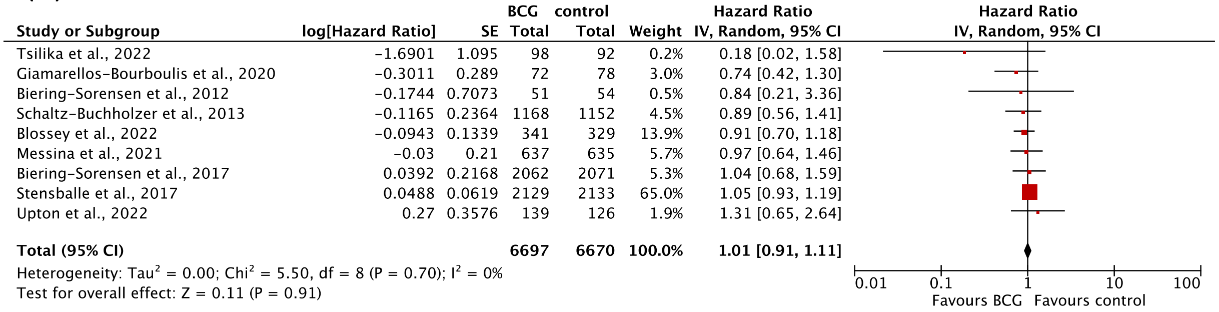

(B)

Study or Subgroup	log[Hazard Ratio]	SE	BCG Total	control Total	Weight	Hazard Ratio IV, Random, 95% CI
Schaltz-Buchholzer et al., 2018	−0.1625	0.1693	3262	3259	15.7%	0.85 [0.61, 1.18]
Messina et al., 2021	−0.03	0.21	637	635	10.2%	0.97 [0.64, 1.46]
Stensballe et al., 2019	−0.0101	0.0778	2129	2133	74.2%	0.99 [0.85, 1.15]
**Total (95% CI)**			**6028**	**6027**	**100.0%**	**0.96 [0.85, 1.10]**

Heterogeneity: $Tau^2 = 0.00$; $Chi^2 = 0.67$, df = 2 (P = 0.72); $I^2 = 0\%$
Test for overall effect: Z = 0.54 (P = 0.59)

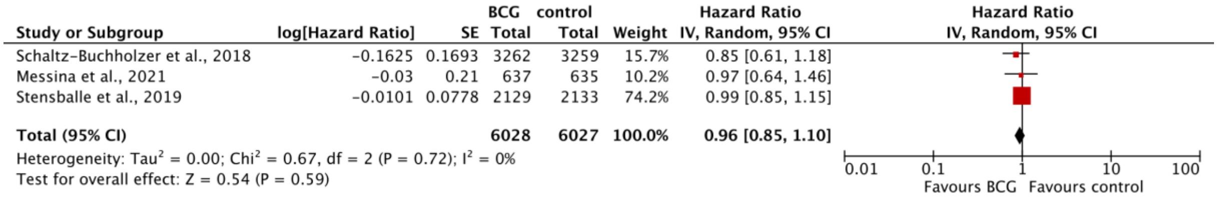

(C)

Study or Subgroup	log[Hazard Ratio]	SE	BCG Total	control Total	Weight	Hazard Ratio IV, Random, 95% CI
Blossey et al., 2022	−1.2379	0.6571	341	329	23.7%	0.29 [0.08, 1.05]
Tsilika et al., 2022	−0.755	0.6966	98	92	22.3%	0.47 [0.12, 1.84]
Schaltz-Buchholzer et al., 2018	−0.7133	0.604	3297	3286	25.7%	0.49 [0.15, 1.60]
Upton et al., 2022	0.6931	0.543	139	126	28.3%	2.00 [0.69, 5.80]
**Total (95% CI)**			**3875**	**3833**	**100.0%**	**0.64 [0.27, 1.53]**

Heterogeneity: $Tau^2 = 0.41$; $Chi^2 = 6.20$, df = 3 (P = 0.10); $I^2 = 52\%$
Test for overall effect: Z = 1.00 (P = 0.32)

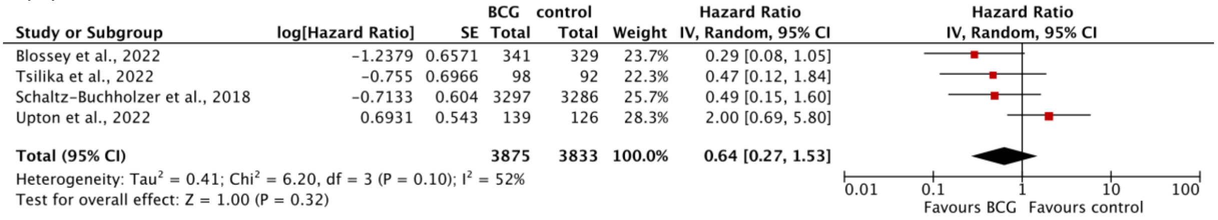

Figure S3. Forest plots of random-effects meta-analysis of BCG trials for (A) hospitalization (B) hospitalization for infections (C) hospitalization for respiratory infections. Solid squares represent hazard ratio estimates for the single studies. The size of the squares represents the weight assigned to the individual study in the meta-analysis and is proportional to the inverse variance (IV) of the estimate. Horizontal lines indicate 95% confidence intervals (CI). The diamond shows the 95% CI for the pooled hazard ratios. Values smaller than 1.0 indicate hazard ratios that favour BCG. BCG = Bacillus Calmette-Guérin, SE = standard error
