# Supplementary material for: Non-Specific Effects of Bacillus Calmette-Guérin: A Systematic Review and Meta-Analysis of Randomized Controlled Trials
Source: Vaccines (Basel). 2023 Jan 4;11(1):121. doi: 10.3390/vaccines11010121 (PMC9866113; doi:10.3390/vaccines11010121)
Supplement: Supplementary file 1 [file vaccines-11-00121-s001.zip › vaccines-2097950-Supplementary Tables.pdf]

Table S1. Electronic datasources for systematic literature search.

Datasource	Website
Pubmed	https://pubmed.ncbi.nlm.nih.gov
Ovid	www.ovid.com
Embase	embase.com
Cochrane CENTRAL	www.cochranelibrary.com
Living Evidence on COVID-19 (plattform not updated since Feb 2022)	https://ispmbern.github.io/covid-19/living-review/
Cochrane COVID-19 Study Register	https://covid-19.cochrane.org/
clinicaltrials.gov	www.clinicaltrials.gov
WHO International Clinical Trials Registry Platform	https://apps.who.int/trialsearch/Default.aspx

Table S2. Detailed search strategy used in this review.

Pubmed	(tuberculosis vaccines[mh] OR bcg vaccine[mh] OR (aeras 402[tiab] OR ((antituberculosis[tiab] OR tuberculosis[tiab] OR tubercle bacilli[tiab]) AND (vaccin*[tiab] OR vacin*[tiab] OR vaksin*[tiab])) OR ((BCG[tiab] OR Calmette Guerin[tiab] OR Calmette*[tiab]) AND<br>(vaccin*[tiab] OR vacin*[tiab] OR vaksin*[tiab] OR live[tiab] OR test[tiab] OR vacin*[tiab] OR bacillus[tiab] OR mycobacterium[tiab] OR copenhagen 1331[tiab])) OR immun bcg pasteur[tiab] OR immuno bcg pasteur[tiab] OR mva 85a[tiab] OR mva85a[tiab] OR mycobax[tiab] OR pastimmun[tiab] OR vpm[tiab] 1002[tiab] OR vpm1002[tiab])) AND (Respiratory Tract Infections[mh] OR COVID-19[mh] OR (((respiratory[tiab] OR respiration[tiab] OR airway[tiab] OR bronchopulmonary[tiab] OR pulmonary[tiab] OR pulmonal[tiab] OR lung[tiab] OR lungs[tiab] OR coronavir*[tiab]) AND (infect*[tiab] OR inflammat*[tiab] OR disease*[tiab] OR disorder*[tiab] OR illness*[tiab] OR syndrom*[tiab])) OR pneumoni*[tiab] OR covid 19[tiab] OR sars cov 2[tiab] OR 2019 ncov[tiab] OR ((new[tiab] OR novel[tiab] OR pandemic[tiab] OR epidemic[tiab]) AND (coronavirus*[tiab] OR corona virus*[tiab])) OR ((non-specific[tiab] OR nonspecific[tiab] OR heterologous[tiab]) AND (effect[tiab] OR effects[tiab])))) AND (randomized controlled trial[pt] OR controlled clinical trial[pt] OR random*[tiab] OR placebo[tiab] OR drug therapy[sh] OR randomly[tiab] OR trial[tiab] OR groups[tiab] NOT (animals [mh] NOT humans [mh]))<br><br>Filters applied: From 2011 to 2022
Medline (Ovid)	(tuberculosis vaccines/ or bcg vaccine/ or (aeras 402 OR ((antituberculosis OR tuberculosis OR tubercle bacilli) ADJ3 (vaccin* OR vacin* OR vaksin*)) OR ((BCG OR Calmette Guerin OR Calmette*) ADJ3 (vaccin* OR vacin* OR vaksin* OR live OR test OR vacin* OR bacillus OR mycobacterium OR copenhagen 1331)) OR immun bcg pasteur OR mva 85a OR mva85a OR mycobax OR pastimmun OR vpm 1002 OR vpm1002).ab,ti,kf) AND (exp Respiratory Tract Infections/ OR COVID-19/ or (((respiratory OR respiration OR airway OR bronchopulmonary OR pulmonary OR pulmonal OR lung OR lungs OR coronavir*) ADJ3 (infect* OR inflammat* OR disease* OR disorder* OR illness* OR syndrom*)) OR pneumoni* OR covid 19 OR (sars ADJ2 cov 2) OR 2019 ncov OR ((new OR novel OR pandemic OR epidemic) AND (coronavirus* OR corona virus*)) OR ((non-specific OR nonspecific OR heterologous) ADJ3 (effect*))).ab,ti,kf) AND (exp Controlled clinical trial/ OR "Double-Blind Method"/ OR "Single-Blind Method"/ OR "Random Allocation"/ OR (random* OR factorial* OR crossover* OR (cross ADJ over*) OR placebo* OR ((doubl* OR singl*) ADJ blind*) OR assign* OR allocat* OR volunteer* OR trial OR groups OR rct).ab,ti.) AND not (exp animals/ NOT humans/) limit 12 to yr="2011 - 2022"
Embase	('BCG vaccine'/de OR 'BCG vaccination'/de OR ('aeras 402' OR ((antituberculosis OR tuberculosis OR 'tubercle bacilli') NEAR/3 (vaccin* OR vacin* OR vaksin*)) OR ((b.c.g. OR BCG OR 'Calmette Guerin' OR Calmette*) NEAR/3 (vaccin* OR vacin* OR vaksin* OR live OR test OR vacin* OR bacillus OR mycobacterium OR 'copenhagen 1331')) OR 'immun bcg pasteur' OR 'mva 85a' OR mva85a OR mycobax OR pastimmun OR 'vpm 1002' OR vpm1002):ab,ti,kw) AND ('respiratory tract infection'/exp OR 'respiratory infections'/de OR 'Coronavirus infection'/exp OR 'covid 19'/exp OR 'coronavirus disease 2019'/exp OR (((respiratory OR respiration OR airway OR bronchopulmonary OR pulmonary OR pulmonal OR lung OR lungs OR coronavir*) NEAR/3 (infect* OR inflammat* OR disease* OR disorder* OR illness* OR syndrom*)) OR pneumoni* OR 'covid 19' OR (sars NEAR/2 'cov 2') OR '2019 ncov' OR ((new OR novel OR pandemic OR epidemic) AND<br>(coronavirus* OR 'corona virus*')) OR ((non-specific OR nonspecific OR heterologous) NEAR/3 (effect*))):ab,ti,kw) AND ('clinical trial'/exp OR 'randomization'/de OR 'Crossover procedure'/de OR 'Double-blind procedure'/de OR 'Single-blind procedure'/de OR (random* OR factorial* OR crossover* OR (cross NEXT/1 over*) OR placebo* OR ((doubl* OR singl*) NEXT/1 blind*) OR assign* OR allocat* OR volunteer* OR trial OR groups OR rct):ab,ti) NOT ([animals]/lim NOT [humans]/lim) AND [2011-2022]/py
Cochrane CENTRAL	("aeras 402" OR ((antituberculosis OR tuberculosis OR "tubercle bacilli") NEAR/3 (vaccin* OR vacin* OR vaksin*)) OR ((b.c.g. OR BCG OR "Calmette Guerin" OR Calmette*) NEAR/3 (vaccin* OR vacin* OR vaksin* OR live OR test OR vacin* OR bacillus OR mycobacterium OR<br>"copenhagen 1331")) OR "immun bcg pasteur" OR "mva 85a" OR mva85a OR mycobax OR pastimmun OR "vpm 1002" OR vpm1002):ab,ti,kw AND (((respiratory OR respiration OR airway OR bronchopulmonary OR pulmonary OR pulmonal OR lung OR lungs OR coronavir*) NEAR/3 (infect* OR inflammat* OR disease* OR disorder* OR illness* OR syndrom*)) OR pneumoni* OR "covid 19" OR (sars NEAR/2 "cov 2") OR "2019 ncov" OR ((new OR novel OR pandemic OR epidemic) AND (coronavirus* OR corona NEXT virus*)) OR ((non-specific OR nonspecific OR heterologous) NEAR/3 (effect*))):ab,ti,kw<br><br>Year range: 2011-2022
Cochrane COVID-19 Study Register	BCG OR Calmette* OR VPM1002<br><br>Limit: from date 01 /01 /2011 – 04 / 07 / 2022

ClinTrials.gov	(BCG OR Calmette OR VPM1002 OR "vpm 1002" OR "copenhagen 1331" OR "immun bcg pasteur" OR "mva 85a" OR mva85a OR mycobax OR pastimmun) AND (respiratory OR respiration OR airway OR bronchopulmonary OR pulmonary OR pulmonal OR pneumonia OR lung OR lungs OR coronavirus OR "corona virus" OR covid OR SARS-Cov-2 OR ncov)
WHO International Clinical trials Registry Platform	(BCG OR Calmette OR VPM1002 OR "vpm 1002" OR "copenhagen 1331" OR "immun bcg pasteur" OR "mva 85a" OR mva85a OR mycobax OR pastimmun)
Living Evidence on COVID-19 database	(BCG) OR (Calmette) OR (VPM1002) OR (mva85a) OR (mycobax) OR (pastimmun)

Table S3. Review checklist with in- and exclusion criteria.

Study Design: Randomized controlled trials
Intervention: Vaccination with Bacillus Calmette-Guérin (BCG)
Outcomes:<br>Primary Outcome: Non-tuberculosis respiratory infections<br>Secondary Outcomes: Non-tuberculosis related, if this information is provided<br>▪ Infections of any origin<br>▪ Sepsis<br>▪ Hospitalizations due to all cause, infectious diseases, respiratory infections, sepsis<br>▪ Mortality due to all cause, infectious diseases, respiratory infections, sepsis
Inclusion criteria:<br>1. RCTs conducted among children and/or adults<br>2. RCTs reporting non-specific effects after BCG vaccination with the outcomes outlined above.<br>3. Date restriction: 2011 – 9.12.2022
Exclusion criteria:<br>1. Studies in animals<br>2. Studies conducted in patients with interfering comorbidities, e.g. bladder cancer<br>3. Studies investigating combinations of interventions<br>4. Case-control studies, cohort and cross-sectional studies<br>5. Systematic review and meta-analysis (just for identification of relevant studies)<br>6. Study protocols
